# Supplementary material for: Suppression of ACE2 SUMOylation protects against SARS-CoV-2 infection through TOLLIP-mediated selective autophagy
Source: Nat Commun. 2022 Sep 3;13:5204. doi: 10.1038/s41467-022-32957-y (PMC9440653; doi:10.1038/s41467-022-32957-y)
Supplement: Supplementary file 3 — Description of Additional Supplementary Files [file 41467_2022_32957_MOESM3_ESM.doc]

**Description of Additional Supplementary Files**

**File name: Supplementary Data 1**

Description: ACE2-ineracting proteins.

**File name: Supplementary Data 2**

Description: Normalized gene counts in 2-D08 treated Calu-3 cells.

**File name: Supplementary Data 3**

Description: Normalized gene counts in *TOLLIP*-deficient Calu-3 cells.
