# Supplementary material for: Suppression of ACE2 SUMOylation protects against SARS-CoV-2 infection through TOLLIP-mediated selective autophagy
Source: Nat Commun. 2022 Sep 3;13:5204. doi: 10.1038/s41467-022-32957-y (PMC9440653; doi:10.1038/s41467-022-32957-y)
Supplement: Supplementary file 7 — Reporting Summary [file 41467_2022_32957_MOESM7_ESM.pdf]

nature research

Corresponding author(s): Shouheng Jin, Jun Cui

Last updated by author(s): Aug 16, 2022

Reporting Summary

Nature Research wishes to improve the reproducibility of the work that we publish. This form provides structure for consistency and transparency in reporting. For further information on Nature Research policies, see our Editorial Policies and the Editorial Policy Checklist.

Statistics

For all statistical analyses, confirm that the following items are present in the figure legend, table legend, main text, or Methods section.

n/a	Confirmed	
☐	☒	The exact sample size (*n*) for each experimental group/condition, given as a discrete number and unit of measurement
☐	☒	A statement on whether measurements were taken from distinct samples or whether the same sample was measured repeatedly
☐	☒	The statistical test(s) used AND whether they are one- or two-sided<br>*Only common tests should be described solely by name; describe more complex techniques in the Methods section.*
☒	☐	A description of all covariates tested
☒	☐	A description of any assumptions or corrections, such as tests of normality and adjustment for multiple comparisons
☐	☒	A full description of the statistical parameters including central tendency (e.g. means) or other basic estimates (e.g. regression coefficient) AND variation (e.g. standard deviation) or associated estimates of uncertainty (e.g. confidence intervals)
☐	☒	For null hypothesis testing, the test statistic (e.g. *F*, *t*, *r*) with confidence intervals, effect sizes, degrees of freedom and *P* value noted<br>*Give P values as exact values whenever suitable.*
☒	☐	For Bayesian analysis, information on the choice of priors and Markov chain Monte Carlo settings
☒	☐	For hierarchical and complex designs, identification of the appropriate level for tests and full reporting of outcomes
☒	☐	Estimates of effect sizes (e.g. Cohen's *d*, Pearson's *r*), indicating how they were calculated

Our web collection on statistics for biologists contains articles on many of the points above.

Software and code

Policy information about availability of computer code

Data collection	For western blotting, ChemiDoc XRS+ System was used for protein detection (Bio-Rad). Confocal images were obtained using a microscope (TCS SP8 STED 3X; Leica). Immunofluorescence staining images were examined using a microscope (Eclipse Ni-E; Nikon). qPCR data were acquired using a LightCycler480 II Real-Time Fluorescence Quantitative PCR System (Roche). The tandem mass spectrometry was performed by Q Exactive mass spectrometer (Thermo Fisher, California, USA).
Data analysis	Image Lab software version 6.0 (Bio-Rad, Hercules, USA); ImageJ software version 1.52 (National Institutes of Health, Maryland, USA); GraphPad Prism software version 8.0 (GraphPad, California, USA); Proteome Discoverer software version 1.4 (Thermo Scientific, Waltham, MA); GPS-SUMO version 2.0; SUMOplot analysis (Abgent) software; JASSA (Joined advanced SUMOylation site and SIM analyser) version 4; Ron Hay's SUMO consensus motif search tool.

For manuscripts utilizing custom algorithms or software that are central to the research but not yet described in published literature, software must be made available to editors and reviewers. We strongly encourage code deposition in a community repository (e.g. GitHub). See the Nature Research guidelines for submitting code & software for further information.

Data

Policy information about availability of data

All manuscripts must include a data availability statement. This statement should provide the following information, where applicable:

- Accession codes, unique identifiers, or web links for publicly available datasets
- A list of figures that have associated raw data
- A description of any restrictions on data availability

All data supporting the findings of this study are available within the paper and its supplementary information files. The mass spectrometry proteomics data have been deposited to the ProteomeXchange Consortium via PRIDE partner repository with the dataset identifier PXD034168 [http://

proteomecentral.proteomexchange.org/cgi/GetDataset?ID=PXD034168]. The RNA-seq data have been deposited in public Gene Expression Omnibus (GEO) database under the accession number GSE171130 [https://www.ncbi.nlm.nih.gov/geo/query/acc.cgi?acc=GSE171130]. The whole-genome sequence (WGS) of SARS-CoV-2 isolate used in this study can be found with GenBank accession number MT123290. Source data are provided with this paper.

Field-specific reporting

Please select the one below that is the best fit for your research. If you are not sure, read the appropriate sections before making your selection.

- [x] Life sciences
- [ ] Behavioural & social sciences
- [ ] Ecological, evolutionary & environmental sciences

For a reference copy of the document with all sections, see nature.com/documents/nr-reporting-summary-flat.pdf

Life sciences study design

All studies must disclose on these points even when the disclosure is negative.

Sample size	Sample size for each experiment is indicated in the legend. No statistical methods were used to predetermine sample sizes. Sample size was chosen based on previous experiments and comparable. The reference of cellular experiments sample size is: Jin, S.H. et al. Tetherin suppresses type I interferon signaling by targeting MAVS for NDP52-mediated selective autophagic degradation in human cells. Mol. Cell 68, 308-322 (2017).
Data exclusions	No exclusion of data was made.
Replication	All experimental findings were reproduced in multiple independent experiments. For each figure, the number of independent experiments or biological replicates is indicated in the figure legends. Western blot and microscopy pictures are from a representative experiment and the number of independent repeats is clearly indicated in the figure legends.
Randomization	For cell culture experiments, cells were split, plated in culture vessels, and then treated with DMSO or drugs. Because control and treatment groups were derived from the same cell line, no randomization could be performed. In animal study, mice were randomly allocated for each group.
Blinding	Investigators were blinded to group allocation during data collection. Investigators were blinded for analysis of immunofluorescence staining. In experiments without subjective estimation like western blotting and qPCR, investigators were unblinded since no bias would be introduced by the investigators.

Reporting for specific materials, systems and methods

We require information from authors about some types of materials, experimental systems and methods used in many studies. Here, indicate whether each material, system or method listed is relevant to your study. If you are not sure if a list item applies to your research, read the appropriate section before selecting a response.

Materials & experimental systems

n/a	Involved in the study	
	x	Antibodies
	x	Eukaryotic cell lines
x		Palaeontology and archaeology
	x	Animals and other organisms
	x	Human research participants
x		Clinical data
x		Dual use research of concern

Methods

n/a	Involved in the study	
x		ChIP-seq
x		Flow cytometry
x		MRI-based neuroimaging

Antibodies

Antibodies used	Horseradish peroxidase anti-Flag (M2; #A8592) and anti-β-actin (#A1978) were purchased from Sigma-Aldrich. Horseradish peroxidase anti-hemagglutinin (HA; #12013819001) and anti-c-myc (Myc; #11814150001) were purchased from Roche Applied Science. Anti-ACE2 (#21115-1-AP), anti-PIAS4 (#14242-1-AP), anti-FIP200/RB1CC1 (#17250-1-AP), anti-Beclin-1 (#11306-1-AP), anti-ATG5 (#10181-2-AP), anti-p62/SQSTM1 (#18420-1-AP), anti-TOLLIP (#11315-1-AP), anti-E-cadherin (#20874-1-AP), anti-α-Tubulin (#11224-1-AP), anti-Syntaxin 6 (#60059-1-Ig), goat anti-mouse (#SA00001-1) and goat anti-rabbit (#SA00001-2) were acquired from Proteintech. K48-linkage specific polyubiquitin antibody (#4289), anti-ULK1 (D8H5) [#8054], anti-ATG13 (E1Y9V) [#13468], anti-ACE2 (#4355), anti-EEA1 (C45B10) [#3288] and anti-Rab7 (D95F2) [#9367] were purchased from Cell Signaling Technology. Anti-SENP3 (#sc-133149), anti-SUMO1 (#sc-5308), anti-SUMO2/3/4 (#sc-393144), and anti-SEC61β (#sc-393633) were from Santa Cruz Biotechnology. Anti-CoV2-N (#40588-T62) was acquired from Sino Biological. Goat anti-rabbit IgG (H+L) highly cross-adsorbed secondary antibody, Alexa Fluor 488 (#A-11034); goat anti-mouse IgG (H+L) highly cross-adsorbed secondary antibody, Alexa Fluor 488 (#A-11029); goat anti-rabbit IgG (H+L) cross-adsorbed secondary antibody, Alexa Fluor 568 (#A-11011); goat anti-mouse IgG (H +L) highly cross-adsorbed secondary antibody, Alexa Fluor 568 (#A-11004); goat anti-mouse IgG (H+L) cross-adsorbed secondary antibody, Alexa Fluor 594 (#A-11005); and goat anti-mouse IgG (H+L) cross-adsorbed secondary antibody, Alexa Fluor 633 (#A-21050)

were purchased from Invitrogen. All primary antibodies were used at a dilution of 1:1000 for immunoblotting, 1:400 for immunoprecipitating and 1:200 for immunofluorescent staining. All secondary antibodies were used at a dilution of 1:5000 for immunoblotting and 1:400 for immunofluorescent staining.

Validation

All primary antibodies were obtained from indicated commercial vendors with ensured quality. Citations are listed as below:
Anti-Flag (M2; #A8592), anti-β-actin (#A1978), horseradish peroxidase anti-hemagglutinin (HA; #12013819001) and anti-c-myc (Myc; #11814150001) were validated in human through IP/WB: Jin, S.H. et al. USP19 modulates autophagy and antiviral immune responses by deubiquitinating Beclin-1. Embo J. 35, 866-880 (2016).
Anti-ACE2 (#21115-1-AP) was KD validated in human through WB: Li, W. et al. A human long non-coding RNA ALT1 controls the cell cycle of vascular endothelial cells via ACE2 and cyclin D1 pathway. Cellular Physiology and Biochemistry 43, 1152-1167 (2017).
Anti-PIAS4 (#14242-1-AP) was KD validated in human through WB: Despras, E. et al. Rad18-dependent SUMOylation of human specialized DNA polymerase eta is required to prevent under-replicated DNA. Nat. Commun. 7, 1-15 (2016).
Anti-FIP200/RB1CC1 (#17250-1-AP) was KD validated in human through WB: Ge, L. et al. Remodeling of ER-exit sites initiates a membrane supply pathway for autophagosome biogenesis. EMBO Rep. 18, 1586-1603 (2017).
Anti-Beclin-1 (#11306-1-AP), anti-ATG5 (#10181-2-AP) and anti-p62/SQSTM1 (#18420-1-AP) were validated in human through WB: Jin, S.H. et al. Tetherin suppresses type I interferon signaling by targeting MAVS for NDP52-mediated selective autophagic degradation in human cells. Mol. Cell 68, 308-322 (2017).
Anti-TOLLIP (#11315-1-AP) was validated through WB/IF by the manufacturer https://www.ptgcn.com/products/TOLLIP-Antibody-11315-1-AP.htm, and further validated in this manuscript using siRNA knockdown.
Anti-E-cadherin (#20874-1-AP) was KD validated in human through WB: Huang L. et al. E-cadherin involvement in human lens epithelial cell transdifferentiation may be associated with N-cadherin. Mol. Med. Rep. 16, 5031-5035 (2017).
Anti-α-Tubulin (#11224-1-AP) was validated in human through WB: Wang X.B. et al. AMPK Promotes SPOP-Mediated NANOG Degradation to Regulate Prostate Cancer Cell Stemness. Dev. Cell 48, 345-360 (2019).
Anti-Syntaxin 6 (#60059-1-Ig) was validated in human through WB/IHC by the manufacturer https://www.ptgcn.com/products/STX6-Antibody-60059-1-Ig.htm, and further validated in this manuscript using WB.
K48-linkage specific polyubiquitin antibody (#4289) was validated in human through IP/WB: Jena, K.K. et al. TRIM16 controls assembly and degradation of protein aggregates by modulating the p62-NRF2 axis and autophagy. Embo J. 37, 1-23 (2018).
Anti-ULK1 (D8H5) [#8054] was validated in human through WB: Najafov, A. et al. RIPK1 promotes energy sensing by the mTORC1 pathway. Mol. Cell 81, 370-385 (2021).
Anti-ATG13 (E1Y9V) [#13468] was validated in human through WB: Odle, R.I. et al. An mTORC1-to-CDK1 switch maintains autophagy suppression during mitosis. Mol. Cell 77, 228-240 (2020).
Anti-ACE2 (#4355) was validated in mice through WB: Clausen, T.M. et al. SARS-CoV-2 infection depends on cellular heparan sulfate and ACE2. Cell 183, 1043-1057 (2020).
Anti-EEA1 (C45B10) [#3288] was validated in human through WB: Tulpule A. et al. Kinase-mediated RAS signaling via membraneless cytoplasmic protein granules. Cell 184, 2649-2664 (2021).
Anti-Rab7 (D95F2) [#9367] was validated in mice through IF: Lanahan A. et al. The neuropilin 1 cytoplasmic domain is required for VEGF-A-dependent arteriogenesis. Dev. Cell 25, 156-168 (2013).
Anti-SENP3 (#sc-133149) was validated in human through WB/IF: Finkbeiner, E., Haindl, M. & Muller, S. The SUMO system controls nucleolar partitioning of a novel mammalian ribosome biogenesis complex. Embo J. 30, 1067-1078 (2011).
Anti-SUMO1 (#sc-5308) was validated in human through IP/WB: Zhenilo, S. et al. DeSUMOylation switches Kaiso from activator to repressor upon hyperosmotic stress. Cell Death Differ. 25, 1938-1951 (2018).
Anti-SUMO2/3/4 (#sc-393144) was validated in human through IP/WB: Wang, Y.Y. et al. STUB1 is targeted by the SUMO-interacting motif of EBNA1 to maintain Epstein-Barr Virus latency. PLoS Pathog. 16, 1-29 (2020).
Anti-SEC61β (#sc-393633) was validated in human through WB: Wang W. et al. TRIM37, a novel E3 ligase for PEX5-mediated peroxisomal matrix protein import. J. Cell Biol. 216, 2843-2858 (2017).
Anti-CoV2-N (#40588-T62) was validated in human through WB: Wu, Y.X. et al. Main protease of SARS-CoV-2 serves as a bifunctional molecule in restricting type I interferon antiviral signaling. Signal Transduct. Target. Ther. 5, 1812-1814 (2020).
All secondary antibodies used in this study were validated by the manufacturer company. The information of its validation data or citation can be found on the manufacturer website by searching the catalog number.

Eukaryotic cell lines

Policy information about cell lines

Cell line source(s)

HEK293T (#GNHu17), Calu-3 (#TCHu157), HeLa (#TCHu187), A549 (#TCHu150), HepG2 (#TCHu72) and Vero E6 (#GNO17) cells were purchased from National Collection of Authenticated Cell Cultures (Shanghai, China). hUVECs (#GDC0635) were acquired from China Center for Type Culture Collection (Wuhan, China). Human alveolar epithelial cells (AECs) (#3200) were obtained from ScienCell Research Laboratories (San Diego, USA).

Authentication

HEK293T, Calu-3, HeLa, A549, HepG2 and Vero E6 cells were authenticated for STR DNA profiling by National Collection of Authenticated Cell Cultures (Shanghai, China). hUVECs were authenticated for STR DNA profiling by China Center for Type Culture Collection (Wuhan, China), no further authentication performed in the laboratory.

Mycoplasma contamination

These cell lines have been tested for mycoplasma contamination by MycoAler Mycoplasma Detection Kit (R&D Systems, cat. CUL001B) and the results of detection showed that cultured cells were not contaimated by mycoplasma.

Commonly misidentified lines
(See ICLAC register)

There is no any commonly misidentified cell lines in this study.

Animals and other organisms

Policy information about studies involving animals; ARRIVE guidelines recommended for reporting animal research

Laboratory animals	In this study, C57BL/6 transgenic hACE2 mice and wild-type BALB/c mice, both male and female, between the age of 8 to 12 weeks, were purchased from Guangzhou Medical Laboratory Animal Center. Animals were kept and bred in a specific-pathogen free (SPF) environment with standard conditions of temperature (20-26 °C) and humidity (40-70 %) under a strict 12 h light cycle (lights on at 08:00 a.m. and off 08:00 p.m.) at Sun Yat-sen University, approved all the experimental protocols concerning the handing of mice.
Wild animals	No any wild animals were observed during this investigation.
Field-collected samples	No any field-collected samples were collected during this investigation.
Ethics oversight	All procedures were approved by the Institutional Animal Care and Use Committee (IACUC) at Sun Yat-sen University.

Note that full information on the approval of the study protocol must also be provided in the manuscript.

Human research participants

Policy information about studies involving human research participants

Population characteristics	No covariate-relevant population characteristics were used.
Recruitment	Biopsy specimens of nasal mucosa were obtained from patients with chronic rhinosinusitis (CRS) who underwent functional endoscopic surgery at the First Affiliated Hospital, Sun Yat-sen University, Guangzhou, China. None of the patients had other systemic diseases and did not receive glucocorticoids, antibiotics, or both within 3 months before the study. All patients provided written informed consent, in accordance with the Declaration of Helsinki and as recommended by the First Affiliated Hospital, Sun Yat-sen University. There is no potential bias in this study as there is no different treatments or groups, all samples are used for human nasal epithelial cell culture.
Ethics oversight	Approval to conduct this study was obtained from the Institutional Review Board of The First Affiliated Hospital, Sun Yat-sen University with ethics approval number [2017]303.

Note that full information on the approval of the study protocol must also be provided in the manuscript.
