# Supplementary material for: Suppression of ACE2 SUMOylation protects against SARS-CoV-2 infection through TOLLIP-mediated selective autophagy
Source: Nat Commun. 2022 Sep 3;13:5204. doi: 10.1038/s41467-022-32957-y (PMC9440653; doi:10.1038/s41467-022-32957-y)
Supplement: Supplementary file 1 [file 41467_2022_32957_MOESM1_ESM.pdf]

Supplementary Information

Suppression of ACE2 SUMOylation protects against SARS-CoV-2 infection through TOLLIP-mediated selective autophagy

Jin *et al.*

Inventory of Supporting Information

1. **Supplementary Figures and figure legends**

   Supplementary Fig. 1 ACE2 can be conjugated with SUMO3.

   Supplementary Fig. 2 SUMOylation inhibitor suppresses the expression of antiviral host genes as well as SARS-CoV-2 infection.

   Supplementary Fig. 3 Dynamic SUMOylation affects ACE2 stabilization.

   Supplementary Fig. 4 SARS-CoV-2 infection drives the degradation and cellular trafficking of ACE2.

   Supplementary Fig. 5 ACE2 undergoes autophagic degradation.

   Supplementary Fig. 6 SUMOylation inhibits ACE2 degradation through TOLLIP-mediated selective autophagy.

   Supplementary Fig. 7 SUMO3 conjugation decreases the K48-linked ubiquitination of ACE2.

2. **Supplementary Table 1** Inhibitors used in this study.
3. **Supplementary Table 2** Sequences of primers for point mutation in this study.
4. **Supplementary Table 3** Sequences of siRNA targets used in this study.
5. **Supplementary Table 4** Sequences of sgRNA targets in this study.
6. **Supplementary Table 5** Sequences of primers for qPCR analysis of this study.

1. Supplementary Figures and figure legends.

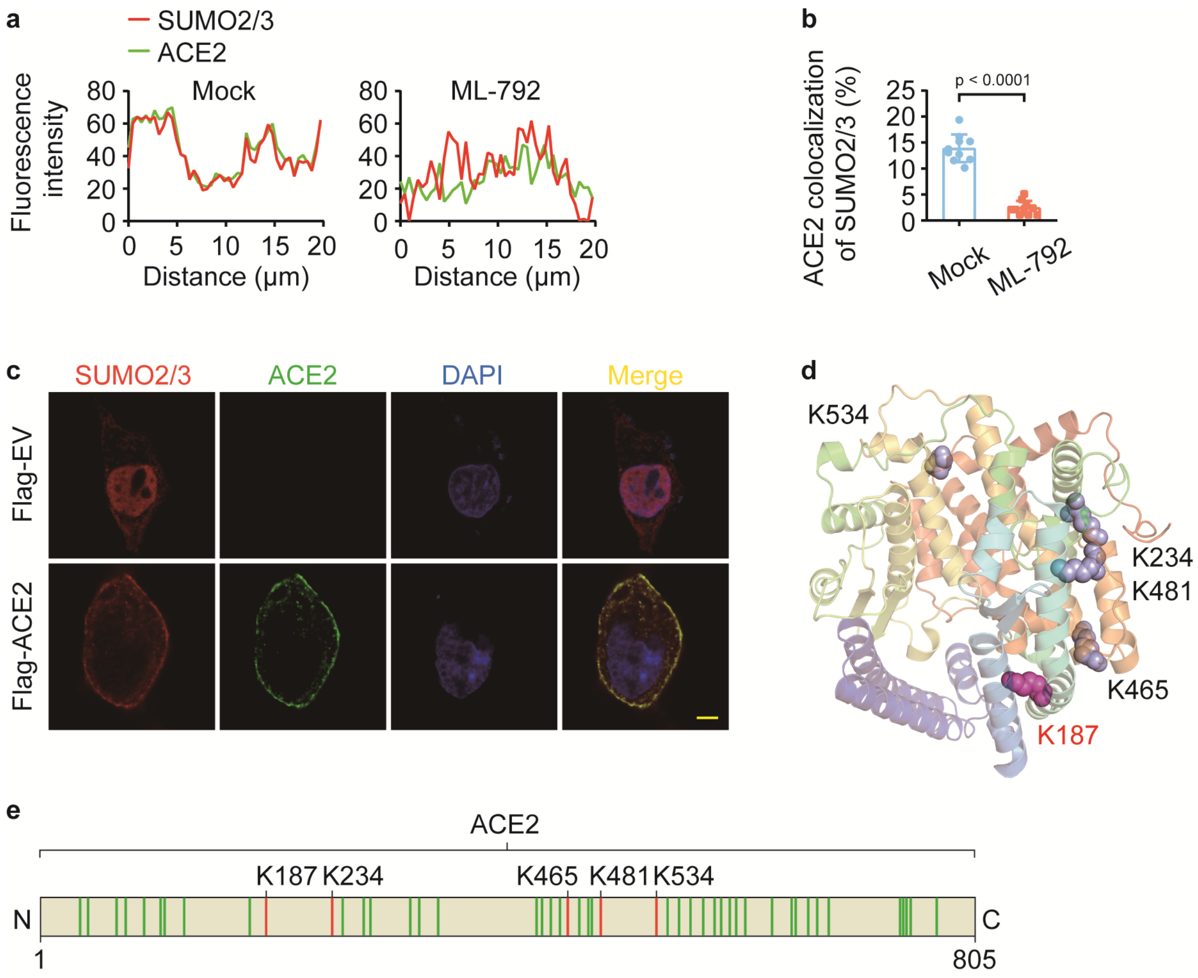

Supplementary Fig. 1 | ACE2 can be conjugated with SUMO3. (**a**) Histogram shows the fluorescence intensity of ACE2 and SUMO2/3 along the white segment in the merged fluorescence panel of Fig. 1g. (**b**) Quantitative analysis of the similar samples as Fig. 1g. (**c**) HeLa cells transfected with empty vector or plasmid encoding ACE2, followed by labeling of ACE2 (green) and SUMO2/3 (red) with specific antibodies. Scale bar, 20 μm. A representative experiment out of three is shown. (**d**) Lysine sites of human ACE2. (**e**) Structure of human ACE2 depicting the identified SUMO motifs. The predicted lysines shown in Fig. 1h are highlighted as purple. The structural information of ACE2 was downloaded from Protein Data Bank (ID: 1R42). In **b**, all error bars, mean values ± SD, *P*-values are

indicated by two-tailed unpaired Student's *t* test (n = 10 cells per group). Source data are provided as a Source data file.

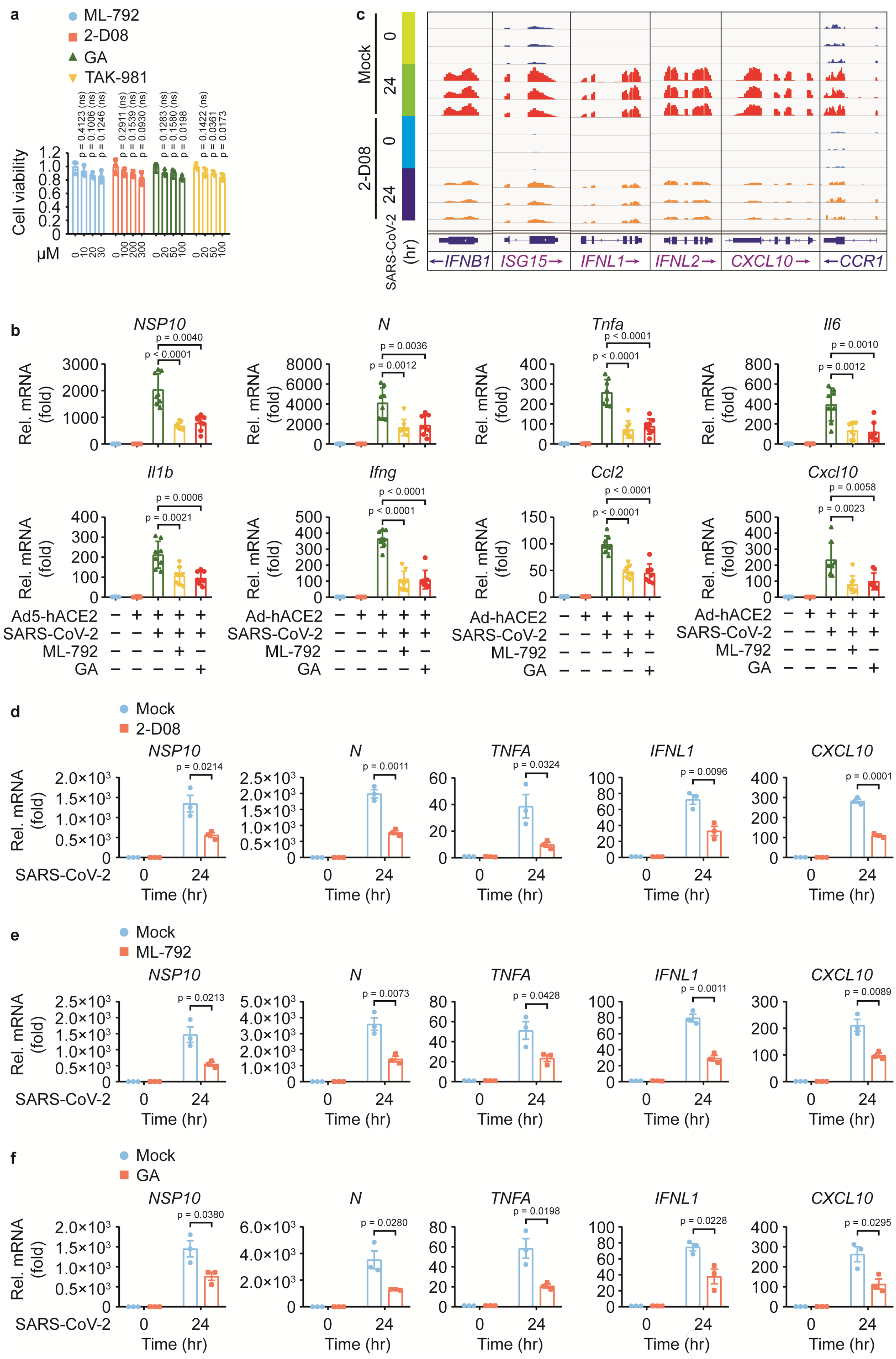
a
ML-792
2-D08
GA
TAK-981
p = 0.4123 (ns)
p = 0.1006 (ns)
p = 0.1246 (ns)
p = 0.2911 (ns)
p = 0.1539 (ns)
p = 0.0930 (ns)
p = 0.1283 (ns)
p = 0.1580 (ns)
p = 0.0198
p = 0.1422 (ns)
p = 0.0361
p = 0.0173
Cell viability
μM
c
Mock
2-D08
SARS-CoV-2 (hr)
IFNB1
ISG15
IFNL1
IFNL2
CXCL10
CCR1
b
NSP10
N
Tnfa
Il6
Il1b
Ifng
Ccl2
Cxcl10
Rel. mRNA (fold)
Ad5-hACE2
Ad-hACE2
SARS-CoV-2
ML-792
GA
d
Mock
2-D08
NSP10
N
TNFA
IFNL1
CXCL10
Rel. mRNA (fold)
SARS-CoV-2
Time (hr)
e
Mock
ML-792
f
Mock
GA

Supplementary Fig. 2 | SUMOylation inhibitor suppresses the expression of antiviral host genes as well as SARS-CoV-2 infection. (**a**) Calu-3 cell viability measured by the LDH assay when incubated with different concentrations of SUMOylation inhibitors for 24 hr. (**b**) qPCR analysis of selected genes of lung homogenates of Ad5-hACE2-transduced BALB/c mice intranasally infected with 1×10^5 PFU of SARS-CoV-2 with or without ML-792 (5 mg/kg) or ginkgolic acid (GA) (2 mg/kg) treatment. (**c**) IGV browser tracks showing the RNA-seq signals of selected genes in Calu-3 cells under SARS-CoV-2 infection for 24 hr together with 2-D08 (100 μM) treatment. (**d**–**f**) qPCR analysis of selected genes of Calu-3 cells challenged with SARS-CoV-2 at MOI = 0.5 with or without 2-D08 (100 μM) (**d**), ML-792 (10 μM) (**e**), or ginkgolic acid (GA) (5 μM) (**f**) treatment. In **a** and **d**–**f**, all error bars, mean values ± SEM, *P*-values are determined by unpaired two-tailed Student's *t* test of n = 3 independent biological experiments. In **b**, all error bars, means values ± SD, *P*-values are determined by unpaired two-tailed Student's *t* test (n = 8 independent biological mice per group). Source data are provided as a Source data file.

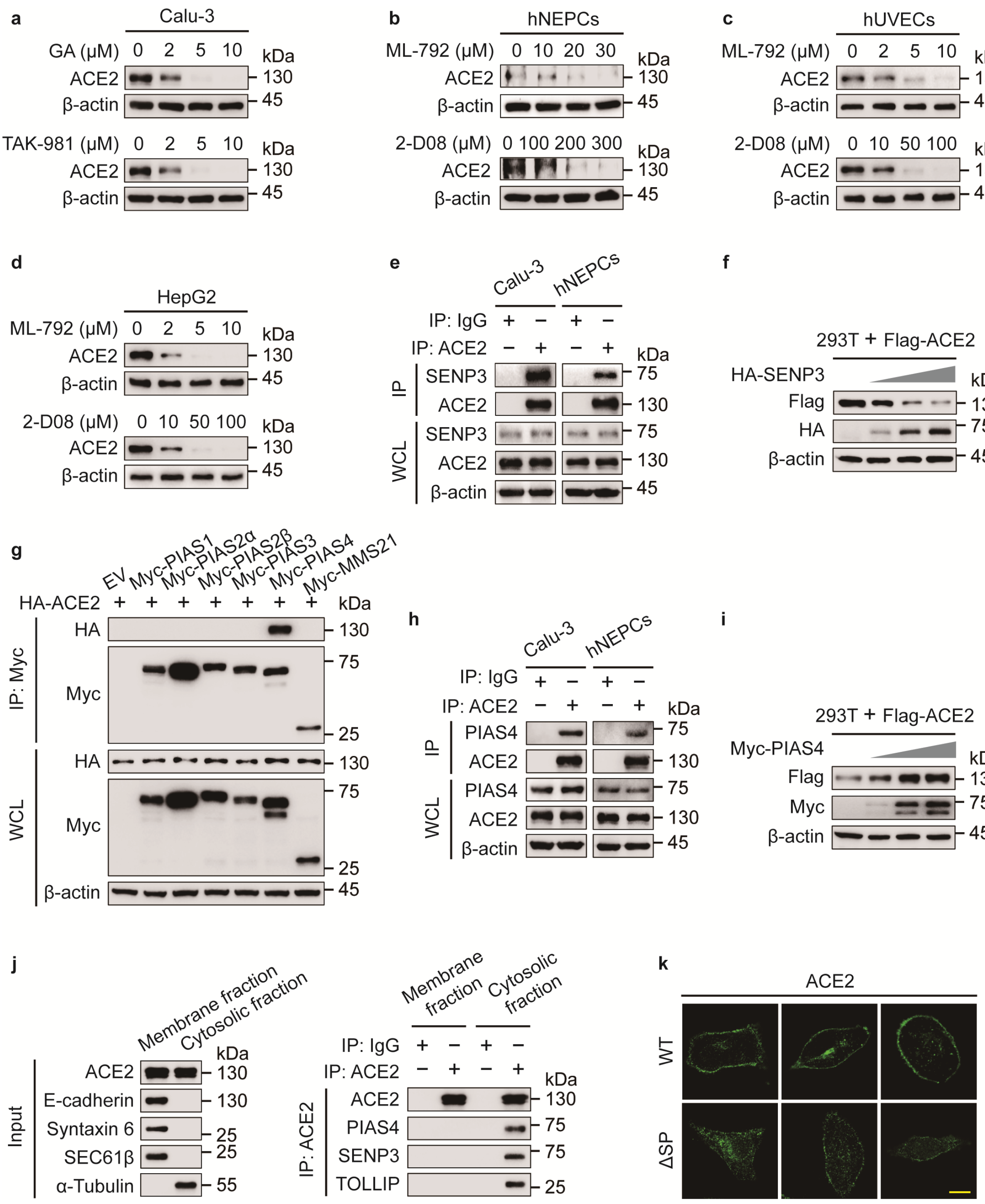

Supplementary Fig. 3 | Dynamic SUMOylation affects ACE2 stabilization. (a) Immunoblot analysis of Calu-3 cells treated with indicated concentration of ginkgolic acid (GA) (up) or TAK-981 (down) for 24 hr. (**b**) hNEPCs were treated with indicated concentrations of ML-792 (up) or 2-D08 (down) for 24 hr, and the protein extracts were harvested for immunoblot analysis. (**c**) Immunoblot analysis of hUVECs treated with

indicated concentrations of ML-792 (up) or 2-D08 (down) for 24 hr. (**d**) HepG2 cells were treated with indicated concentrations of ML-792 (up) or 2-D08 (down) for 24 hr, and the protein extracts were harvested for immunoblot analysis. (**e**) Extracts of Calu-3 cells (left) and hNEPCs (right) were immunoprecipitated with anti-ACE2 antibody and analyzed with immunoblotting. (**f**) Immunoblot analysis of extracts of 293T cells transfected with expression vector for Flag-ACE2 and increasing doses of expression vector for HA-SENP3 (wedge). (**g**) 293T cells were transfected with vectors encoding HA-ACE2 and indicated Myc-tagged SUMO E3 ligases, followed by immunoprecipitation with anti-Myc beads and immunoblot analysis with anti-HA. (**h**) Extracts of Calu-3 cells (left) and hNEPCs (right) were subjected to immunoprecipitation and immunoblot analysis with indicated antibodies. (**i**) Immunoblot analysis of extracts of 293T cells transfected with expression vector for Flag-ACE2 and increasing doses of expression vector for Myc-PIAS4 (wedge). (**j**) Membrane and cytosolic fractions of Calu-3 cells were immunoblotted with antibodies directed against ACE2, a cytosol marker (α-tubulin), an ER membrane marker (SEC61β), a Golgi apparatus membrane marker (Syntaxin 6), or a PM marker (E-cadherin). The membrane and cytosolic fractions were immunoprecipitated with anti-ACE2 antibody and analyzed with immunoblotting. (**k**) HeLa cells transfected with plasmids encoding WT and the ΔSP mutant form of ACE2, followed by labeling of ACE2 (green) with specific antibodies. Representative confocal microscopy images from n = 3 independent biological samples for each group. Scale bar, 20 μm. For **a**–**j**, similar results are obtained by three independent biological experiments. Source data are provided as a Source data file.

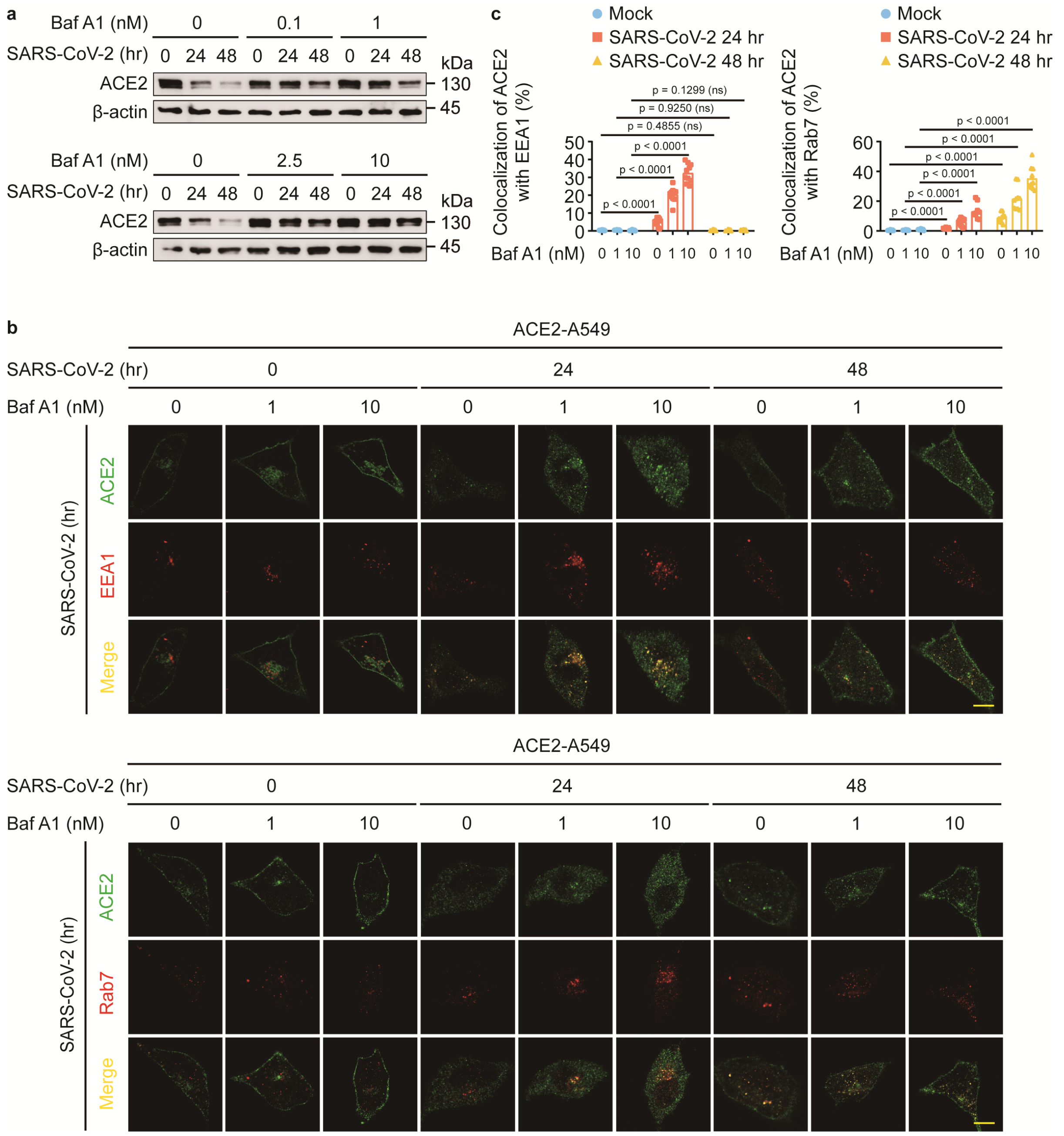

Supplementary Fig. 4 | SARS-CoV-2 infection drives the degradation and cellular trafficking of ACE2. (**a**) Calu-3 cells treated with different concentrations of Baf A1 were challenged with SARS-CoV-2 (MOI = 0.5) for indicated time points. The protein extracts were harvested for immunoblot analysis. (**b**) Confocal microscopy of ACE2-expressing A549 cells treated with Baf A1 (1 or 10 nM) were infected with SARS-CoV-2 (MOI = 0.5) for indicated time points, followed by labeling of ACE2 (green) and EEA1 (red) or Rab7 (red)

with specific antibodies. Scale bar, 20 μm. A representative experiment out of three is shown. (**c**) Quantitative analysis of the similar samples as (**b**). In **c**, all error bars, mean values ± SD, *P*-values are indicated by two-tailed unpaired Student's *t* test (n = 10 cells per group). For **a**, similar results are obtained by three independent biological experiments. Source data are provided as a Source data file.

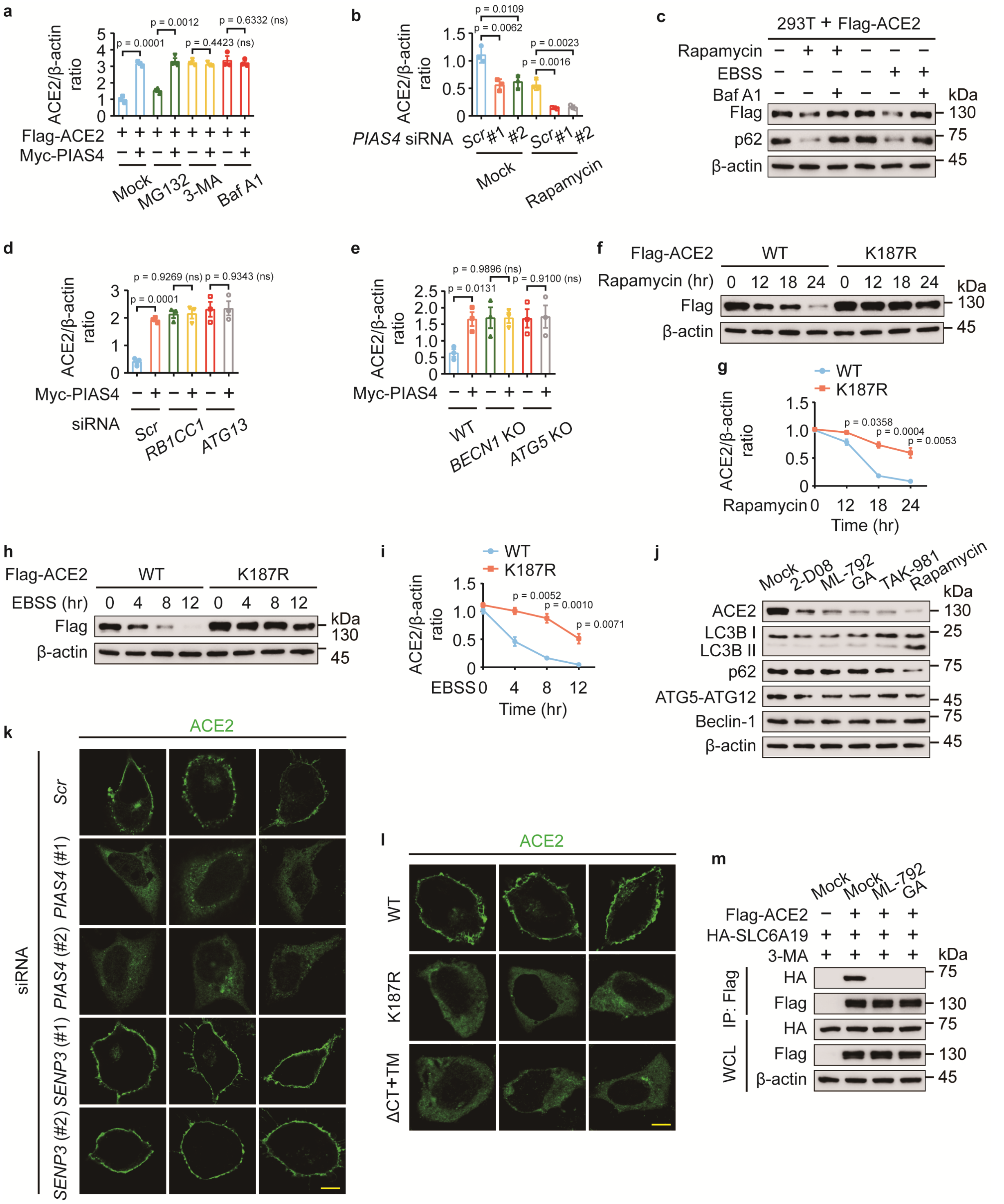

Supplementary Fig. 5 | ACE2 undergoes autophagic degradation. (**a**) The quantification of ACE2 expression from Fig. 4a. (**b**) The quantification of ACE2 expression from Fig. 4b. (**c**) 293T cells transfected with Flag-ACE2 vector were treated with rapamycin (250 nM) for 24 hr or cultured with EBSS for 12 hr, in the presence or absence of bafilomycin A1 (0.2 μM) for

6 hr. The lysates were analyzed with immunoblotting. (**d**) The quantification of ACE2 expression from Fig. 4d. (**e**) The quantification of ACE2 expression from Fig. 4e. (**f**, **g**) Immunoblot analysis of 293T cells transfected with Flag-ACE2 (WT or K187R) and treated with rapamycin (250 nM) for indicated time points, with quantification shown in (**g**). (**h**, **i**) Immunoblot analysis of 293T cells transfected with Flag-ACE2 (WT or K187R) and cultured within EBSS for indicated time points, with quantification shown in (**i**). (**j**) Immunoblot analysis of Calu-3 cells treated with 2-D08 (200 μM), ML-792 (10 μM), GA (5 μM), TAK-981 (5 μM), or rapamycin (250 nM). (**k**) HeLa cells transfected with scramble, *PIAS4*-specific, or *SENP3*-specific siRNAs together with plasmid expressing Flag-ACE2, followed by labeling of ACE2 (green) with specific antibodies. Representative confocal microscopy images from n = 3 independent biological samples for each group. Scale bar, 20 μm. (**l**) HeLa cells transfected with expressing vector of Flag-ACE2 or its mutants, followed by labeling of ACE2 (green) with specific antibodies. Representative confocal microscopy images from n = 3 independent biological samples for each group. Scale bar, 20 μm. (**m**) 293T cell were transfected with Flag-ACE2 and HA-SLC6A19 and treated with ML-792 (10 μM) or ginkgolic acid (GA) (5 μM) in the presence of 3-MA (10 mM) for 6 hr. The lysates were harvested for coimmunoprecipitation and immunoblot analysis. In **a**, **b**, **d**, **e**, **g** and **i**, all error bars, mean values ± SEM, *P*-values are determined by unpaired two-tailed Student's *t* test of n = 3 independent biological experiments. For **c**, **f**, **h**, **j** and **m**, similar results are obtained by three independent biological experiments. Source data are provided as a Source data file.

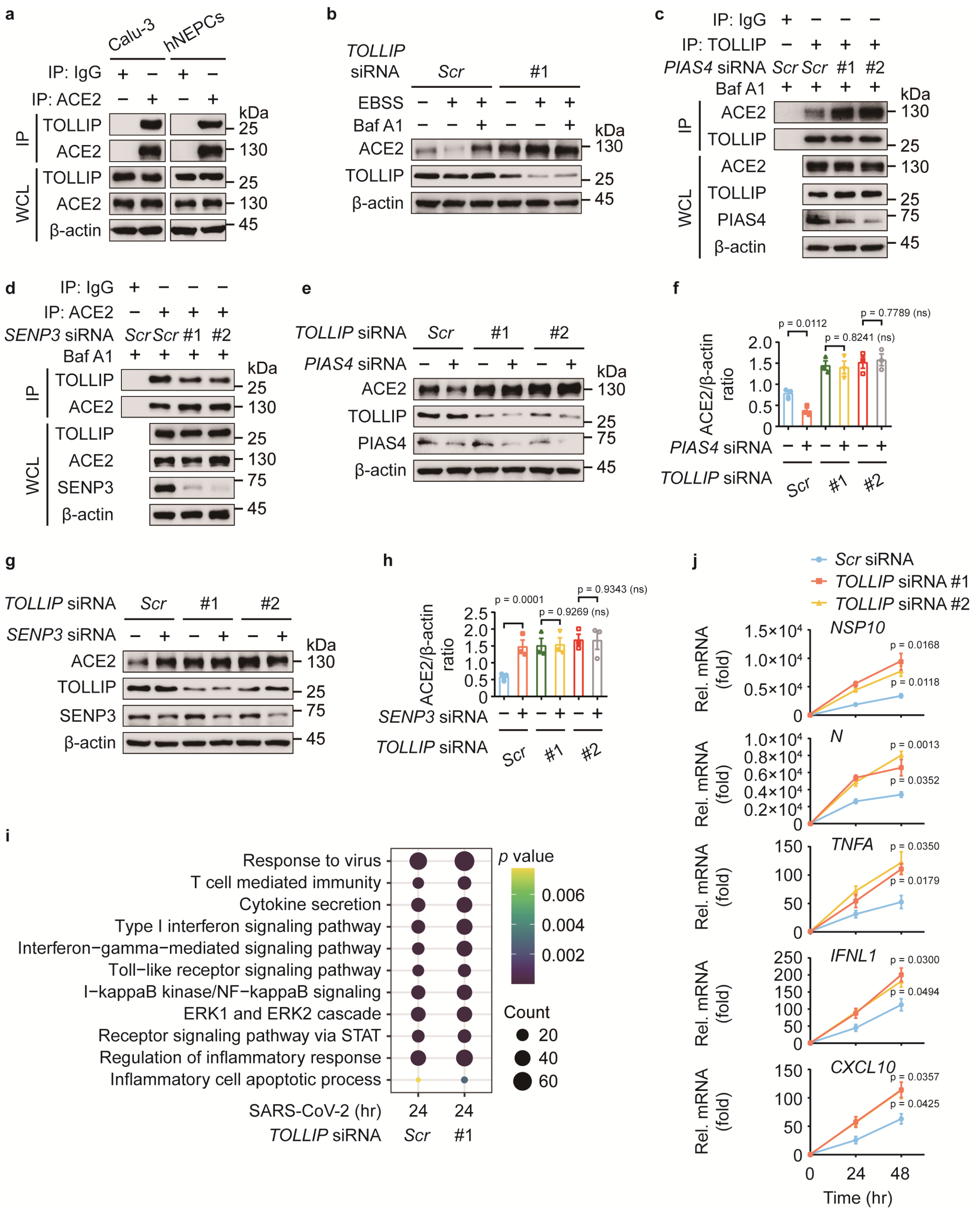

Supplementary Fig. 6 | SUMOylation inhibits ACE2 degradation through TOLLIP-mediated selective autophagy. (**a**) Extracts of Calu-3 cells (left) and hNEPCs (right) were subjected to immunoprecipitation and immunoblot analysis. (**b**) Immunoblot analysis of Calu-3 cells transfected with scramble or *TOLLIP*-specific siRNAs and cultured in

EBSS for 12 hr together with or without Baf A1 (0.2 μM) treatment. (**c**, **d**) Lysates of Calu-3 cells transfected with scramble siRNA as well as *PIAS4*-specific (**c**) or *SENP3*-specific (**d**) siRNA were immunoprecipitated and analyzed with immunoblotting using indicated antibodies. (**e**, **f**) Immunoblot analysis of Calu-3 cells transfected with scramble or *TOLLIP*-specific siRNAs for 8 hr, followed by *PIAS4*-specific siRNA transfection, with quantification shown in (**f**). (**g**, **h**) Immunoblot analysis of Calu-3 cells transfected with scramble or *TOLLIP*-specific siRNAs for 8 hr, followed by *SENP3*-specific siRNA transfection, with quantification shown in (**h**). (**i**) Dot-plot visualization of enriched GO terms showing the enriched genes up-regulated by SARS-CoV-2 infection with or without transfection of *TOLLIP*-specific siRNA comparing with negative control. Calu-3 cells were infected with SARS-CoV-2 (MOI = 0.5) for 24 hr. The ordinate is the GO term description. Bubble size represents the number of DEGs in GO classification; the enrichment *P*-value is calculated by Fisher exact test; and different color represents different *P*-value. (**j**) Calu-3 cells were transfected with scramble or *TOLLIP*-specific siRNAs treated with SARS-CoV-2 (MOI = 0.5) for indicated time points. Relative expression levels of selected genes were measured by qPCR. In **f**, **h** and **j**, all error bars, mean values ± SEM, *P*-values are determined by unpaired two-tailed Student's *t* test of n = 3 independent biological experiments. For **a**–**e** and **g**, similar results are obtained by three independent biological experiments. Source data are provided as a Source data file.

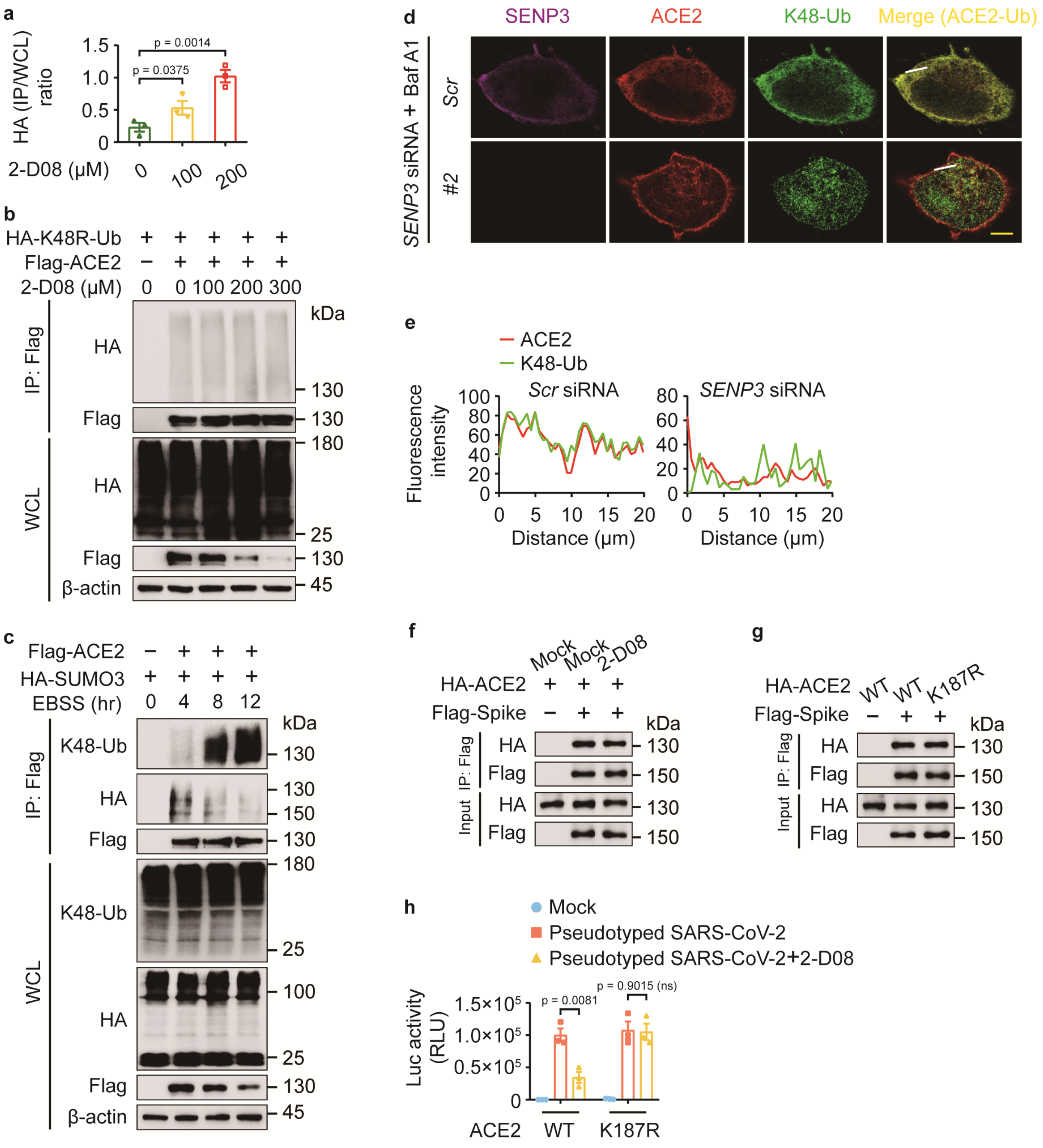

Supplementary Fig. 7 | SUMO3 conjugation decreases the K48-linked ubiquitination of ACE2. (**a**) The quantification of immunoprecipitated ACE2 from Fig. 6a. (**b**) Immunoprecipitation and immunoblot analysis of 293T cells transfected with plasmids expressing Flag-ACE2 and HA-tagged ubiquitin (Ub) K48R mutant with indicated concentrations of 2-D08 treatment for 24 hr. (**c**) 293T cells transfected with vectors expressing Flag-ACE2 and HA-SUMO3 were cultured in EBSS for indicated time points. The

lysates were harvested for immunoprecipitation with anti-Flag beads and immunoblot analysis with indicated antibodies. (**d**) HeLa cells transfected with scramble or *SENP3*-specific siRNAs and then transfected with Flag-ACE2 and HA-K48-linked ubiquitin (Ub) plasmids, followed by labeling of SENP3 (purple), ACE2 (red) and K48-Ub (green) with specific antibodies. Scale bar, 20 μm. A representative experiment out of three is shown. (**e**) Histogram shows the fluorescence intensity of ACE2 and K48-Ub along the white segment in the merged fluorescence panel of (**d**). (**f**) Flag-Spike of SARS-CoV-2 was expressed in 293T cells, purified using Flag affinity column, and eluted with Flag peptide. HA-ACE2 purified from 293T cells in the absence or presence of 2-D08 (200 μM) using HA affinity column was eluted with HA peptide. Purified HA-ACE2 was incubated with immunopurified Flag-Spike in reaction buffer *in vitro*. After pull-down with Flag-beads, the bound material was analyzed by immunoblotting. (**g**) Flag-Spike and HA-ACE2 (WT or K187R) purified from 293T cells were incubated in reaction buffer *in vitro*, and then immunoprecipitated and analyzed by immunoblotting. (**h**) Luciferase activity of 293T cells transfected with plasmid expressing WT or K187R mutant form of ACE2, along with SARS-CoV-2 S pseudotyped virus infection for 48 hr in the absence or presence of 2-D08 (200 μM). In **a** and **h**, all error bars, mean values ± SEM, *P*-values are determined by unpaired two-tailed Student's *t* test of n = 3 independent biological experiments. For **b**, **c**, **f** and **g**, similar results are obtained by three independent biological experiments. Source data are provided as a Source data file.

2. Supplementary Table 1 | Inhibitors used in this study.

Compound name	Formula	Function
2-D08	$C_{15}H_{10}O_5$	2-D08 inhibits SUMOylation by preventing transfer of SUMO from the UBC9-SUMO thioester to the substrate.
ML-792	$C_{21}H_{23}BrN_6O_5S$	ML-792 selectively blocks SAE enzyme activity and total SUMOylation.
Ginkgolic acid (GA)	$C_{22}H_{34}O_3$	Ginkgolic acid inhibits protein SUMOylation by blocking formation of the E1-SUMO intermediate.
MG-132	$C_{26}H_{41}N_3O_5$	MG-132 effectively blocks the proteolytic activity of the 26S proteasome complex.
3-MA	$C_6H_7N_5$	3-MA is a widely used inhibitor of autophagy via its inhibitory effect on class III PI3K.
Baf A1	$C_{35}H_{58}O_9$	Baf A1 blocks autophagosome-lysosome fusion and inhibits acidification and protein degradation in lysosomes of cultured cells.
Rapamycin	$C_{51}H_{79}NO_{13}$	Rapamycin binds to FKBP12 and specifically acts as an allosteric inhibitor of mTORC1.
Chlorpromazine (CPZ)	$C_{17}H_{19}ClN_2S$	CPZ causes clathrin lattices to assemble on endosomal membranes and prevents the assembly of coated pits at the cell surface.
Methyl-β-cyclodextrin (MβCD)	$C_{54}H_{94}O_{35}$	MβCD can deplete cholesterol from cell membranes.
Cytochalasin D (CD)	$C_{30}H_{37}NO_6$	Cytochalasin D inhibits the G-actin–cofilin interaction by binding to G-actin. Cytochalasin D also inhibits the binding of cofilin to F-actin and decreases the rate of both actin polymerization and depolymerization in living cells.
TAK-981	$C_{25}H_{28}ClN_5O_5S_2$	TAK-981 covalently binds to the small ubiquitin-like modifier protein, forming an adduct with SUMO protein. This prevents the transfer of SUMO from the SAE to UBC9.

3. Supplementary Table 2 | Sequences of primers for point mutation in this study.

Gene	**Primers**
ACE2 (K187R)	**Fwd (5′ to 3′)**: AGAGTATGTGGTCTTGAGAAATGAGATGGCAAGAG
	**Rev (5′ to 3′)**: CTCTTGCCATCTCATTTCTCAAGACCACATACTCT
ACE2 (K234R)	**Fwd (5′ to 3′)**: TACCTTTGAAGAGATTAGACCATTATATGAACATC
	**Rev (5′ to 3′)**: GATGTTCATATAATGGTCTAATCTCTTCAAAGGTA
ACE2 (K465R)	**Fwd (5′ to 3′)**: GAGGTGGATGGTCTTTAGAGGGGAAATTCCCAAAG
	**Rev (5′ to 3′)**: CTTTGGGAATTTCCCCTCTAAAGACCATCCACCTC
ACE2 (K481R)	**Fwd (5′ to 3′)**: AAAGTGGTGGGAGATGAGGCGAGAGATAGTTGGGG
	**Rev (5′ to 3′)**: CCCCAACTATCTCTCGCCTCATCTCCCACCACTTT
ACE2 (K534R)	**Fwd (5′ to 3′)**: ACTTTGTCAAGCAGCTAGACATGAAGGCCCTCTGC
	**Rev (5′ to 3′)**: GCAGAGGGCCTTCATGTCTAGCTGCTTGACAAAGT

4. Supplementary Table 3 | Sequences of siRNA targets used in this study.

**Name**	**Sequence (5' to 3')**
Scramble (*Scr*) siRNA	GUGAGCGUCUAUAUACCAUTT
SENP3 siRNA #1	ACGUGGACAUCUUCAAUAATT
SENP3 siRNA #2	CAAUAAGGAGCUACUGCUATT
PIAS4 siRNA #1	GGAGUAAGAGUGGACUGAATT
PIAS4 siRNA #2	GCUCUACGGAAAGUACUUATT
RB1CC1 siRNA #1	UGUCGUCUCCUAAUCCUAUAATT
RB1CC1 siRNA #2	GCAAAGAAAUUAGGGAAUCUUTT
ATG13 siRNA #1	GGACCUUCUAUCGGGAGUUUCTT
ATG13 siRNA #2	GACCUGGACAAGUUUAUUAAATT
TOLLIP siRNA #1	CACACAAUGGCGCCAAGAAUCTT
TOLLIP siRNA #2	CGACUGAACAUCACGGUGGUATT

5. Supplementary Table 4 | Sequences of sgRNA targets in this study.

**Name**	**Sequence (5' to 3')**
SENP1 target	TCGGTCCAAATGTCCTTGCC
SENP2 target	GCACCGACCGGTCGCAGAAA
SENP3 target	GTGGCGTCGCACCCCCTTGC
SENP5 target	GGACCATCAAGAGACCCGTA
SENP6 target	GTAAGGTTAAGTCGGCTCCA
SENP7 target	GAGTCTAGTATAAGAATACA
SENP8 target	GGTCCATCTTGTACTGACGA

6. Supplementary Table 5 | Sequences of primers for qPCR analysis of this study.

Gene	**Fwd** (5' to 3')	**Rev** (5' to 3')
human *RPL13A*	GCCATCGTGGCTAAACAGGTA	GTTGGTGTTCATCCGCTTGC
human *PIAS4*	TCAGATGCTCCTGGGTTTCGTG	TTCTTCTTGGCGTAGCGGGTC
human *ACE2*	CTTTCCGTCTGAATGACAACA	CACTATCACTCCCATCACAACTC
human *TNFA*	CCTCTCTCTAATCAGCCCTCTG	GAGGACCTGGGAGTAGATGAG
human *IFNL1*	GTGACTTTGGTGCTAGGCTTG	GCCTCAGGTCCCAATTCCC
human *CXCL10*	GTGGCATTCAAGGAGTAGCTC	GCCTTCGATTCTTGGATTCAG
mouse *Gapdh*	GAAGGGCTCATGACCACAGT	GGATGCAGGGATGATGTTCT
mouse *Cxcl10*	CCTGCCCACGTGTTGAGAT	TGATGGTCTTAGATTCCGGATTC
mouse *Ifng*	AAAGACAATCAGGCCATCAGC	CCTCAAACTTGGCAATACTCA
mouse *Ilb*	GCAACTGTTCCTGAACTCAACT	ATCTTTTGGGGTCCGTCAACT
mouse *Il6*	TAGTCCTTCCTACCCCAATTTCC	TTGGTCCTTAGCCACTCCTTC
mouse *Tnfa*	CCCTCACACTCAGATCATCTTCT	GCTACGACGTGGGCTACAG
mouse *Ccl2*	ACCTGCTGCTACTCATTCACC	AAACTACAGCTTCTTTGGGACA
SARS-CoV-2 *NSP10*	CCCTGTGGGTTTTACACTTAA	ACGATTGTGCATCAGCTGA
SARS-CoV-2 *N*	GGGGAACTTCTCCTGCTAGAAT	CAGACATTTTGCTCTCAAGCTG
